# Impact of an electronic medical record-based automated screening program for critical congenital heart disease: Emirates Health Services, United Arab Emirates

**DOI:** 10.1186/s12911-022-01900-y

**Published:** 2022-06-21

**Authors:** Sumaya Al Zarouni, Noor Majed Al Mheiri, Kalthoom Al Blooshi, Yousif Al Serkal, Neema Preman, Sadaf Ahsan Naqvi, Yasir Khan

**Affiliations:** 1 Emirates Health Services, Dubai, United Arab Emirates; 2 Cerner Middle East, Dubai, United Arab Emirates

**Keywords:** Critical congenital heart disease, Electronic medical record, New-born screening

## Abstract

**Background:**

Almost eight children per 1000 live births are expected to have a congenital heart defect globally. The seven most critical congenital heart conditions that cause severe compromise on the patient’s quality and duration of life are collectively referred to as the Critical Congenital Heart Diseases (CCHD). CCHD is a critical condition that requires prompt detection and intervention as a life-saving measure. *Pulse oximetry* is a non-invasive, highly specific, and moderately sensitive method that can be used for screening new-borns for CCHD. The Emirates Health Services (EHS), UAE, adopted a strategy of developing a stringent program for newborn screening of Critical Congenital Heart disease, which would help in the early diagnosis and treatment of CCHD patients. An automated EMR (Wareed) driven solution was introduced to enhance this program as part of the routine workflow for the nurse care providers.

**Methods:**

Our study is a retrospective observational study that aims to understand: the prevalence of CCHD in our target population and to study the impact of an automated program on screening compliance and its implications for early diagnosis of CCHD.

**Results:**

We found that an EMR-driven automated screening program was highly effective in achieving high compliance (98.9%). It created a (statistically significant) improvement in the disease identification for CCHD in live births at EHS facilities.

**Conclusion:**

We conclude that implementing an automated protocol through the EMR can effectively improve new-born screening coverage. It reduces the days to CCHD diagnosis, which would improve health outcomes in neonates.

## Introduction

Congenital Heart Disease (CHD) refers to a group of structural or functional heart diseases that present at birth. It is typically defined as “a gross structural abnormality of the heart or intrathoracic great vessels that is actually or potentially of functional significance” [1]. The spectrum of disease severity varies from mild/clinically undetectable symptoms to severe life-threatening conditions [2]. Almost 8 children per 1000 live births are expected to have a congenital heart defect globally [2, 3] in the United States, it is estimated that approximately 4.2% of the total neonatal deaths are contributed by this condition [4–7]. CHDs may vary in their clinical implications—the seven most critical congenital heart conditions that cause severe compromise on the patient’s quality and duration of life are collectively referred to as the critical congenital heart diseases (CCHD). The seven conditions that constitute the CCHD complex are: hypoplastic left heart syndrome, pulmonary atresia (with intact ventricular septum), tetralogy of Fallot, total anomalous pulmonary venous return, transposition of the great arteries, tricuspid atresia, and truncus arteriosus. Studies from the United States indicate that approximately 4800 babies are born with a CCHD every year; out of these, 280 get discharged with an unrecognized CCHD [8]. CCHD is associated with hypoxemia, which may be present in 17–31% of all CHDs [9, 10]. Even if it is clinically silent in the initial stage, presentation with hypoxemia can make it possible to detect CCHD at an earlier stage. As indicated by its name, the CCHD is a critical condition that requires prompt detection and intervention as a life-saving measure. These interventions can range from surgical correction to catheterization before the first birthday [11]. Children discharged from the hospital without a diagnosis of CCHD at the time of birth are at a greater risk of clinical deterioration and adverse outcomes [12]. This disease should be detected right after birth to hasten the care process. Since Hypoxemia is one of the earliest clinical signs, research proves that pulse oximetry can be effectively utilized for the initial screening of CCHDs that cause hypoxemia [13]. Pulse oximetry is a non-invasive, highly specific, and moderately sensitive method of detecting hypoxemia in new-borns at 24 h. While cyanosis may not be apparent clinically, pulse oximetry can effectively detect dropping blood oxygen levels. To increase the accuracy of this screening, it is recommended to repeat the screening test two times in neonates with low oxygen levels before declaring them as failed for pulse oximetry. While the causes for low blood oxygen in a neonate can be other than CCHD, all the potential causes require prompt clinical attention [14].

With the advancement in technology and increasing capabilities of Healthcare Information systems, it has been recognized that EMR can play a pivotal role in creating efficiencies and improving healthcare outcomes [15]. Clinical decision support integrated within the EMR leads to improved quality of care, as has been proved by many studies previously [16, 17]. This concept has been applied to various new-born screening protocols globally, and it was found to be very helpful in elevating the quality of care [18].

The Emirates Health Services (EHS) UAE governs a network of hospitals covering Dubai and the Northern Emirates of the country. With the vision of making the UAE a hub for world-class healthcare [19], the EHS adopted a strategy of developing a stringent program for new-born screening of Critical Congenital Heart disease, which would help in the early diagnosis and treatment of CCHD patients. Reports from the UAE indicate congenital heart disease prevalence to be around 1% of total births [20]. According to studies from the Middle Eastern population, the cause-specific mortality from CCHD in children under one year of age is estimated to be 211.7 per 100,000 population [21]. American Academy of Pediatrics has laid down discreet guidelines for new-born screening for congenital heart diseases [22]. EHS incorporated these guidelines in their policies in 2011 as a part of their new-born screening program. To further enhance this program, the guidelines were implemented as an automated EMR (Wareed) driven workflow in 2018–2019. CCHD screening of new-borns at EHS facilities became a part of the routine workflow for the nurse care providers [23]. EHS EMR, Wareed makes it possible to utilize smart logics and rules in automating the order for CCHD screening for every live birth within our network, reducing dependency on physician orders.

This paper discusses an automated program for CCHD screening of all new-borns in EHS and shares the findings from our CCHD screening data extracted through the Wareed database. We analyze the impact of this new screening program on the early identification of CCHD in children born within EHS facilities.

Our study aims to evaluate the impact of an EMR-driven automated CCHD screening program on screening coverage and initiation of CCHD management in new-borns across all EHS hospitals.

Additionally, we extracted EMR driven data to analyze the demographic and clinical variables in the EHS hospital birth cohort of 2016, 2017, 2019 and 2020.

## Methods

Utilizing best practice guidelines, EHS developed a policy regarding CCHD screening for all new borns at their hospitals. The process involved the development of detailed guidelines, designing a streamlined clinical workflow outlining roles and responsibilities, followed by procurement of recommended pulse oximetry devices, and staff training on the process. To support this entire process and to apply it successfully, the EMR was utilized as a medium to incorporate and facilitate this change. An EMR solution was designed with a thorough focus on workflow requirements. Clinical decision support was incorporated within the solution design of this EMR program to standardize practice, reduce gaps and delays in screening, and minimize reaction time if a baby fails the screening. Data was captured through the EMR to analyze the operational as well as clinical requirements of this project.

### Workflow

A well thought, seamless workflow was designed and embedded within EMR to ensure maximum utilization of this solution by EHS clinicians. As soon as a live birth is documented within the EMR, the system triggers an auto-order utilizing smart logics and rules to conduct CCHD screening on the neonate through pulse oximetry. This is generated as a task for the nursing staff responsible for providing care to the new-born. The initial screening should be done 24 h after birth or right before discharge if the patient is discharged within 24 h of birth.

If a patient has low oxygen saturation on initial screening, the system detects this abnormality and generates another auto-order for the second screening and subsequently for a third screening in case the saturations are low on the second attempt as well.

**Fig. 1:**
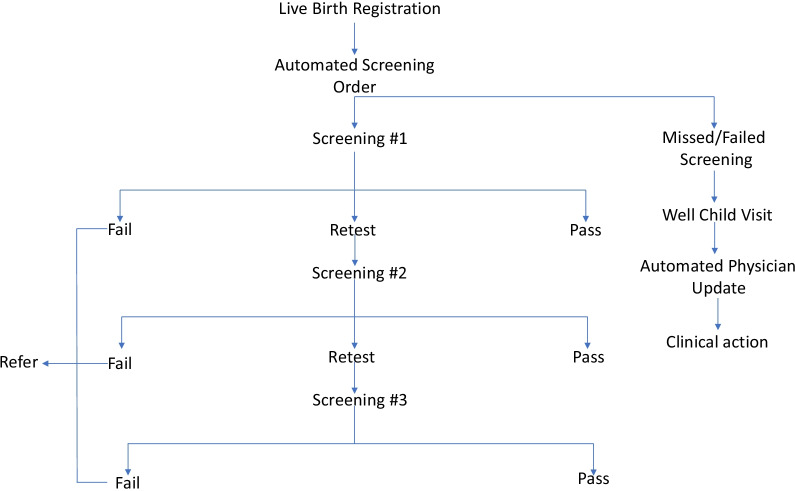
Workflow for initial and subsequent CCHD screening for all live births at EHS hospitals

If a child sustains low oxygenation even at the third screening, the child is declared to have failed CCHD screening, and an auto-order set is activated through the system to investigate further and confirm the cause of low oxygenation. This includes alerting physicians and triggering referral orders for escalated assessment protocol. To address the new-borns who might have missed CCHD screening at the birth encounter, the EMR displays new-born screening results for all children presenting for their well-child visits in outpatient clinics. This serves as decision support for physicians to identify children who missed or failed the screening and take further clinical action. This is an EMR driven, automated workflow that reduces dependency on manual ordering as well as interpretation; it also provides prompt decision support and guides the clinicians for further action as per the guidelines (Fig. 1).

#### Interpretation of findings

*Pass*

Child maintaining appropriate pulse oxygen levels (95% or higher) at screening.

*Fail*

A child with a pulse oxygen level between 90–94% after three repeated screenings.

*OR*

A child with a pulse oxygen level of 89% or below in one screening.

*Not appropriate*

In some scenarios, users were allowed to not conduct the screening by indicating that they did consider the screening for the child but, due to some reason, decided against it. This included babies immediately shifted to NICU after delivery or cases where equipment malfunction made it impossible to conduct the screening. The user indicated that an attempt was indeed made in all these cases.

Having this category allowed us to differentiate from cases where there was no screening attempt at all.

*Not performed*

This represents cases where no documentation of CCHD screening was performed or offered on a new-born’s record.

### Study design

This is a retrospective, observational study that was conducted on the entire live birth record within the EMR to evaluate the CCHD screening compliance, overall data distribution, trends, and the average age of diagnosis for CCHD in the birth Cohort of all the children born at the EHS hospitals in 2019 and 2020 (a comparison made to 2016 and 2017 where applicable).

### Study setting

Emirates Health Services, UAE governs a network of 17 hospitals spread over Dubai and the Northern Emirates of UAE. 9 out of these hospitals have maternity and birth facilities, catering to the pre-natal and post-natal requirements of the mothers and the babies. All birth record data was extracted from these hospitals to be included in our study.

### Inclusion criteria

All live births within EHS hospitals that occurred in 2019 and 2020 are included in our analysis. To compare the duration between birth and confirmed CCHD diagnosis after birth, a similar data set was extracted for all live births in the year 2016 and 2017. (Some hospitals were piloted with CCHD solution in 2018; therefore, 2018 data is not included in our analysis to maintain appropriate matching of the data set and intervention parameters).

### Exclusion criteria

Stillbirths were excluded from our analysis as including stillbirths would not be applicable to our program. For the later phase of analysis (comparing means of days to diagnosis), we excluded those CCHD patients who were born outside the EHS network and presented later, as well as those from the EHS births who did not receive CCHD screening as per 2019 and 2020 birth record, were also excluded from this analysis.

### Plan of analysis

The required data set was extracted from EMR for the years 2016, 2017, 2019, and 2020.

It was then analyzed using Microsoft Excel and SPSS software (version 19).

We analyzed the data for the frequency distribution of general demographic and clinical variables for all four years. We analyzed the percentage compliance of the screening offered to a patient (recorded as Passed, Failed, not appropriate) in the birth cohort post-implementation of the automated program (2019–2020).

Our primary outcome variable was the average time to diagnosis of CCHD from birth and the difference in time to diagnosis between the two birth cohorts (pre and post-program implementation). To evaluate any difference before and after implementation, we applied independent t-test that calculated the difference in the mean time to disease identification.

### Bias elimination

The data were deidentified to eliminate any chances of bias.

The study was approved by the ethical committee of the Ministry of Health and Prevention UAE.

## Results

We collected all relevant data from our EMR database, where the birth cohort of all children born in 2016,2017, 2019, and 2020 was analyzed. This included live births only (as stillbirth does not qualify for our study requirements).

The descriptive distribution of the birth cohort was analyzed along with specific parameters pertaining to the CCHD screening protocol and its outcomes. EHS recorded a (slightly) higher number of male births than females in both years (Table 1).

**Table 1:** Total births in EHS hospitals ( 2016,2017,2019,2020)

Gender	2016	2017	2019	2020
Male	4514	5500	4962	4847
Female	4306	5147	4686	4623
Not documented	3	0	1	2
Total births	8823	10,647	9649	9472

For the birth cohort of 2019 and 2020, we analyzed the adoption of the new program, as compliance with screening protocols was our main output of interest. It was found that CCHD screening was offered to 99% of the new-borns before discharge across all EHS facilities (Table 2).

**Table 2:** CCHD Screening performed for live births at EHS facilities (2019,2020)

Screening performed	2019 Frequency	2019 Percentage	2020 Frequency	2020 Percentage
Yes	9551	98.9	9403	99.27
No	98	1.1	69	0.72
Total	9649	100.0	9472	100.0

We also analyzed the results documented from screening—amongst those screened in 2019 and 2020, almost 95% of children passed their CCHD screening before they were discharged (in both birth cohorts), 0.4% and 0.57% failed it (respectively). In contrast, others were not applicable for the screening (according to the guidelines) (Table 3).

**Table 3:** CCHD Screening results for live births at EHS facilities (2019,2020)

Screening result	2019 Frequency	2019 Percentage	2020 Frequency	2020 Percentage
Failed screening	39	0.4	54	0.57
Not appropriate (not applicable)	377	3.9	300	3.16
Not performed	106	1.1	69	0.72
Passed screening	9127	94.6	9049	95.5
Total	9649	100.0	9472	100.0

Furthermore, a retrospective analysis was conducted on all children born in 2016,2017, 2019, and 2020 and had a diagnosis of CCHD within Wareed. There were 29 children in 2016, 27 in 2017, 29 cases in 2019, and 30 in 2020.

Out of these Children, many were born outside EHS facilities and presented for further treatment of their CCHD much later after birth. Since we aim to measure the effectiveness of the screening program offered right after birth, we excluded births from outside EHS facilities from further analysis. We also excluded a few children born in 2019 and 2020 who were not provided CCHD screening at birth. After these exclusions, we had 22 live births from each cohort. EMR records of these live births were analyzed to determine the first CHD/CCHD diagnosis placed and the mean difference in age of this documentation. We found a statistically significant (*p* = 0.027) difference in the mean age of CCHD identification before and after introducing the automated screening program. There was a mean reduction of almost 24 days between the two groups (Table 4).

**Table 4:** Difference in average age (days) of detection of CCHD in births before and after program implementation

	CCHD (EHS births)	Mean Days to diagnosis	Mean difference in days	*p* Value (95% CI)
Pre-implementation (2016, 2017)	22	39.3		
Post-implementation (2019, 2020)	22	15.5	23.74	0.027

## Discussion

Congenital Heart disease is a public health concern globally, given its burden, potential morbidity, and mortality [24]. Over the past decades, there has been a lot of research on effective management strategies for improving the clinical burden of this condition [14]. It has been recognized that many children get discharged from the hospitals without detection of a CCHD risk which can delay the final diagnosis and treatment for these children [25]. The Emirates Health Services, UAE implemented their CCHD screening program to support the early detection of this life-threatening condition through a non-invasive, low cost, and simple method (of pulse oximetry), as per best practice guidelines [26]. This program has been implemented through their EMR (Wareed), which ensures that the guidelines are standardized and enforced across the entire network.

Our study is an endeavor to understand the dynamics of a comprehensive congenital cardiac disease screening program for new-borns in a large population. We leveraged the EMR data-base to create insights across all EHS facilities. Since the Screening order is an automated order through the EMR, we found that the compliance with screening for all new-borns was higher (99%) than in some other systems around the world [27]. Utilizing EMR to prompt guideline adherence to end-users has proven an effective strategy in many settings [28, 29]. EHS employed the same strategy to implement their CCHD screening protocol. Having a program coverage so high is a huge achievement, and it can serve as a guiding strategy for other countries that are still struggling to achieve improved coverage for their screening programs. There was only 1% of the patients who had no documentation of screening performed, which is explained by the guidelines as the unavoidable fraction of patients who wouldn’t receive due to various reasons ( parents’ refusal, prenatal diagnosis of CCHD, Echocardiogram performed before the screening, etc.) [22]. We can conclude that utilizing smart technological support through the EMR is an effective strategy for implementing a standard practice across our network. The EMR not only guides the clinicians in following EHS workflow, but regular monitoring of the data generated through the system has been a valuable tool for decision-makers to improve the service further.

We found an EHS-wide CCHD prevalence of 0.25–0.3% in live births of both birth cohorts, respectively. We also estimated the average time from birth to the first confirmed diagnosis of CCHD in both the birth cohorts. It is known that failing a CCHD birth screening is not a definitive diagnosis of CCHD, but in fact, a newborn can fail the screening due to several other factors [30, 31]. Due to this reason, a child with failed CCHD screening is referred for further investigations to detect the reason for failure [32, 33].

The most valuable finding of this study is that in our birth cohort, the identification of cardiac disease reduced significantly before and after program implementation. This is a promising finding that supports having an automated CCHD screening program for early detection of this life-threatening condition. This finding strengthens our belief that our strategy has been effective in the universal screening of all new-borns and reducing the time of final diagnosis for all new-borns.

We propose other healthcare systems to utilize their EMR to enforce their screening programs as it can reduce practice variation and eventually contribute to better health outcomes. EMR-induced smart program reduces practice gaps and covers the entire workflow spectrum while making it smart and easy to utilize. We also propose establishing a system of close follow-up for all CCHD survivors, as many studies have directed towards long-lasting health impacts in CHD survivors even in adult life [34–36]. Another area for future research would be to explore and determine maternal and familial factors that can help identify the risk of CCHD in the prenatal period. [37].

## Limitations

Several children diagnosed with CCHD are born outside the EHS network; hence we could not analyze the screening assessment offered to them at birth. This issue is uniform across the two birth cohorts; therefore, we do not expect it to impact the significance of our findings.

### Strengths

Our study includes all live births within EHS, covering the entire cohort within the specified time periods. We utilized EMR-generated data to ensure accuracy and completeness. We share an EMR-driven program implementation strategy that has yielded very high compliance from the end-users, and proven to have significant improvement in disease identification-hence we propose other healthcare systems adopt such a strategy to increase the effectiveness of their CCHD screening program.

## Conclusion

Utilizing EMR as the platform for implementing the screening program and using automated orders can yield high adherence to hospital protocols. Having a well-defined workflow for screening and follow-up of children with potential CHD has a positive impact on the overall identification of CCHD, which would positively impact child health outcomes. Research from other parts of the world created a direction for screening strategies and their outcomes in special populations and different regions of the world. This study aligns with those requirements highlighted by other researchers [14].

## Data Availability

The datasets used and/or analyzed during the current study are available from the corresponding author on reasonable request.
